# Environmental Enrichment Induces Changes in Long-Term Memory for Social Transmission of Food Preference in Aged Mice through a Mechanism Associated with Epigenetic Processes

**DOI:** 10.1155/2018/3725087

**Published:** 2018-07-16

**Authors:** Simona Cintoli, Maria Cristina Cenni, Bruno Pinto, Silvia Morea, Alessandro Sale, Lamberto Maffei, Nicoletta Berardi

**Affiliations:** ^1^Neuroscience Institute, CNR, Via G. Moruzzi 1, 56124 Pisa, Italy; ^2^Department of Neuroscience, Psychology, Drug Research and Child Health (NEUROFARBA), University of Florence, Florence, Italy; ^3^Scuola Normale Superiore, Pisa, Italy

## Abstract

Decline in declarative learning and memory performance is a typical feature of normal aging processes. Exposure of aged animals to an enriched environment (EE) counteracts this decline, an effect correlated with reduction of age-related changes in hippocampal dendritic branching, spine density, neurogenesis, gliogenesis, and neural plasticity, including its epigenetic underpinnings. Declarative memories depend on the medial temporal lobe system, including the hippocampus, for their formation, but, over days to weeks, they become increasingly dependent on other brain regions such as the neocortex and in particular the prefrontal cortex (PFC), a process known as system consolidation. Recently, it has been shown that early tagging of cortical networks is a crucial neurobiological process for remote memory formation and that this tagging involves epigenetic mechanisms in the recipient orbitofrontal (OFC) areas. Whether EE can enhance system consolidation in aged animals has not been tested; in particular, whether the early tagging mechanisms in OFC areas are deficient in aged animals and whether EE can ameliorate them is not known. This study aimed at testing whether EE could affect system consolidation in aged mice using the social transmission of food preference paradigm, which involves an ethologically based form of associative olfactory memory. We found that only EE mice successfully performed the remote memory recall task, showed neuronal activation in OFC, assessed with c-fos immunohistochemistry and early tagging of OFC, assessed with histone H3 acetylation, suggesting a defective system consolidation and early OFC tagging in aged mice which are ameliorated by EE.

## 1. Introduction

Aging of the brain is a complex biological process associated with decline in sensory, motor, and cognitive functions. In particular, a decline in declarative learning and memory performance is a typical feature of the normal aging process. Human and animal model data are in accordance to show that during aging, changes in neuronal morphology and density as well as changes in synaptic density, function, and plasticity are specific to each area of the brain [1–3]; brain areas crucial for declarative memory formation, such as hippocampus and other medial temporal lobe structures, show differential volume decline with age, associated with loss of synaptic density, changes in neuronal electrophysiological properties, deficits in synaptic plasticity [1, 3, 4], and changes in gene transcription [5, 6] and in epigenetic mechanisms, leading to reduction in plasticity factors necessary for the induction and local consolidation of synaptic efficacy changes [7, 8]. Aged rodents have contributed a crucial part of these data [1, 9, 10].

Environmental enrichment (EE) is an experimental protocol classically defined as “a combination of complex inanimate and social stimulation” [11] which provides animals with the opportunity to attain high levels of voluntary physical activity on running wheels and to enhance exploration, cognitive activity, and social interaction. EE causes brain changes at functional, anatomical, and molecular level, including changes in plasticity factors and mechanisms (see [9]), with clear benefits for learning and memory [12–15], particularly evident in aged animals [9, 12, 16]. Most of the studies have been conducted in aged rodents, but EE has been found to provide cognitive benefits also in other aged mammals (see [9]). Positive effects of EE on cognitive processes have been found in young, adult, and aged rodents both for hippocampal-dependent and hippocampal-independent learning and memory [9, 12–22]. For instance, EE improves spatial memory, (see [9, 23, 24]), object recognition memory [9, 24], social novelty [9, 24], and fear memory [25] in aged mice and in mouse models of neurodegeneration (see [26]). These beneficial effects in aged animals have been related to EE action on neurogenesis, neurotrophic factors (BDNF), IGF-I, synaptic plasticity, and neurotransmitter systems (see [9]). In particular, the improvement in declarative learning and memory performance in EE aged rodents has been correlated with EE attenuating the age-related changes in hippocampal dendritic branching, spine density, neurogenesis, gliogenesis, and neural plasticity, including its epigenetic underpinnings [9, 20–22, 26–31].

Declarative memories (memories for facts and events) are not acquired in their definitive form but undergo a gradual process of stabilization over time to allow long-term maintenance [32–34]. In animals, typical protocols to test declarative memory are those testing spatial memory, recognition memory, and in general associative memory. In the process of associative memory formation, consolidation, and maintenance, the hippocampus is believed to integrate, in the form of an anatomical index, information transmitted from distributed cortical networks that support the various features of a whole experience [34], rapidly merging these different features into a coherent memory trace. Consolidation of this new memory trace at the cortical level would then occur slowly via repeated and coordinated reactivation of hippocampal-cortical networks in order to progressively increase the strength and stability of corticocortical connections that represent the original experience. Therefore, these types of memories depend on the medial temporal lobe system, including the hippocampus, for their formation, but, over days to weeks, they become increasingly dependent on other brain regions such as the neocortex and in particular the prefrontal cortex (PFC) [20–23]. This process of time-dependent gradual reorganization of the brain regions supporting remote memory storage is known as systems-level memory consolidation or system consolidation [24–28]. Recently, it has been shown that early tagging of cortical networks is a necessary neurobiological process for remote associative olfactory memory formation using the social transmission of food preference (STFP) paradigm, an ethologically based form of associative olfactory memory [29–31], and that this tagging involves epigenetic mechanisms in the recipient orbitofrontal (OFC) areas [35, 36].

Whether EE can enhance system consolidation in aged animals has not been tested; in particular, whether the early tagging mechanisms in OFC areas demonstrated by Lesburgueres et al. [36] in young animals are deficient in aged animals and whether EE can ameliorate them is not known.

This study aimed at testing whether EE could affect system consolidation in STFP in aged mice. As already pointed out, aged mice are considered a good model of aging, with translation value [10]. For instance, deficits in the same types of memory appear in aged humans and mice, increases in oxidative stress parameters and neuroinflammation, which are typical alterations of the aged brain, are present in both aged humans and mice [9, 10], and, even if amyloid plaques are not found in aged mouse brain, recent work has shown that aged mice do show an increase in amyloid oligomers, which also characterize human aging [37]. Regarding the estimated correspondence between murine and human age, some comparative studies have estimated [38] that senescence begins in mice around eighteen months of age; the age of mice in the present paper (15-16 months) would then correspond, approximately, with the upper limit of human adulthood (65 years).

We have studied whether EE could affect system consolidation in STFP, a protocol particularly suitable to study recent and remote associative memory [39], in aged mice, using the same protocol used by Lesburgueres et al. [36] to demonstrate for the first time a role for histone acetylation in the early tagging of OFC in young animals. We found that only EE mice successfully performed the remote associative olfactory memory recall task and showed neuronal activation in OFC, assessed with c-fos immunohistochemistry. Early tagging of OFC, assessed with histone H3 acetylation [36], was found in EE but not in SC aged mice.

## 2. Materials and Methods

### 2.1. Animal Treatment

Male and female C57BL/6 mice of 14 months of age were used in this study. All the procedures were approved by the Italian Ministry of Health. Animals were housed in the animal house with a 12 h/12 h light/dark cycle and with food and water available ad libitum. At 14 months of age, animals were assigned to one of the following rearing conditions for 40 days: environmental enrichment (EE mice, *n* = 32) or standard condition (SC mice, *n* = 40). SC rearing condition consisted of 26 × 18 × 18 cm cages housing 3 animals per cage. EE rearing condition was achieved using a large cage (44 × 62 × 28 cm) containing several food hoppers, one running wheel for voluntary physical exercise, and differently shaped objects (tunnels, toys, shelters, and stairs) that were repositioned twice a week and completely substituted with others once a week. The rearing conditions were maintained throughout the behavioral tests.

### 2.2. Social Transmission of Food Preference (STFP) Test

For the STFP test, mice, male and female, were transferred in same-sex littermate groups (2–5 animals per box) in Plexiglas cages. The test was performed during the light phase of the cycle. The STFP task is based on food neophobia in rodents. Mice show a preference for eating a food that is cued with an odor previously experienced in the breath of a conspecific, over a flavor cued with a novel scent [40, 41]. In this protocol, “observer” mice interact, during the learning phase, with a “demonstrator” mouse that has recently eaten a novel food. When observer mice are subsequently presented with a choice between the food eaten by the demonstrator and some other novel foods, they prefer the food eaten by the demonstrator, the familiar food. This phenomenon depends on the observer mice detecting olfactory cues in the breath of the demonstrator mouse during their interaction within the learning phase (sniff interaction). The subsequent food preference serves as a measure of memory for those olfactory cues. Before starting the test, one mouse from each cage was designated as the “demonstrator” and the others as the “observers.” For the behavioral experiments we have used a total of 17 EE animals and 23 SC animals: in the EE group, 13 EE animals performed the behavioral tasks as observers and 4 EE mice served as demonstrator mice; in the SC group, 16 SC animals performed the task as observers and and 7 SC mice served as demonstrators.

The STFP task consisted of three distinct phases as described in the protocol of Wrenn et al. [41, 42]. To minimize neophobia during the experiments, mice were habituated to eat powdered rodent chow from 4 oz (113.40 g) glass food jar assemblies (Dyets Inc., Bethlehem, PA). Jars were approximately 7 cm in diameter and 5 cm in depth [41]. The jar assemblies have been selected in order to prevent mice from crawling and digging into the food and spilling the food from the jar.

Before the first phase, the experimenter prepared the food jar assemblies using flavored food and recorded the weights of the jars. Cocoa-flavored chow was obtained by mixing ground cocoa with plain powdered chow to give a 2% (*w*/*w*) cocoa mixture. Cumin-flavored chow was obtained by mixing cumin with plain powdered chow to give a 0.5% (*w*/*w*) cumin mixture [36].

In phase I, the demonstrators were exposed to cocoa-flavored food during a 1 h feeding session in a cage without water. At the end of phase I, the jar assemblies were removed and weighed: the demonstrator that did not eat at least 0.2 g of food was not used in the further steps of the experiment. In phase II, the learning phase, the demonstrators, immediately after the 1 h exposure to the flavored food, were placed into the cages containing their respective observers: mice were allowed to interact freely for 30 min. During phase II, the experimenter constantly watched the demonstrator from a distance of ≅50 cm and recorded the number of times that each “observer” sniffed the muzzle of the demonstrator. A sniff is defined as a close (<2 cm) orientation of the observer's nose toward the front or side of the demonstrator's muzzle [41]. The scoring of observer sniffs of the demonstrator's muzzle can provide critical data showing that the impairment in food preference is not due to changes in social behavior and insufficient social interaction. Phase III, the choice phase, started after a selected retention interval (1 h, 24 h, or 30 days). Each observer was placed in an individual cage with two jars in the opposite side of the cage for counterbalancing the position. One jar contained the familiar food (cocoa-flavored), consumed by the demonstrator observer mice had interacted with, the other one the novel food (cumin-flavored). After 1 h of feeding period, the jars were removed from the cages and weighed; the amount of food eaten from each jar was recorded. The preference for the familiar food over the novel food was taken as an index of memory for the familiar food. For all three phases, the experimenter was blind to the rearing conditions of the mice.

To control for the effects of extra handling, novel food sniffing, and consumption and interaction with the demonstrator mouse on c-fos activation and histone H3 acetylation, we used pseudolearning (PL) mice; these animals experienced arena exploration, jar exploration, food consumption, and interaction with the demonstrator, but no learning was involved. Indeed, PL subjects (12 PL-EE and 12 PL-SC) did not detect any olfactory cue in the breath of the demonstrator mouse during the interaction period, since demonstrator mice (3 EE and 5 SC) did not eat any food before interaction. In this way, we can control for differences in c-fos expression or H3 acetylation simply due to extra handling, exposure to the arena, food, and demonstrator mouse, in the learning group and isolate the effect of learning on c-fos activation and H3 acetylation.

For c-fos-positive cell analysis, we used, at 24 h retention interval, EE (*n* = 4), SC (*n* = 4), PL-EE (*n* = 4), and PL-SC (*n* = 4); at 30-day retention interval, EE (*n* = 4), SC (*n* = 4), PL-EE (*n* = 4), and PL-SC (*n* = 4); for histone H3 acetylation analysis in the OFC, we used EE (*n* = 4), SC (*n* = 4), PL-EE (*n* = 4), and PL-SC (*n* = 4).

A possible confounding factor is the presence of an innate flavor preference for one of the flavors used in the experiment. Over the years, several different flavorants have been successfully used in the basic procedure of STFP [43]. The pairs of flavorant used in the present paper and their specific concentrations were based on literature data [36] and on pilot data indicating that by giving naïve mice a choice between the two flavors used in our experiment, mice ate the same amount of each flavored food in the absence of STFP training (*n* = 5, paired *t*-test cocoa-flavored versus cumin-flavored *p* = 0.359).

### 2.3. Immunohistochemistry

We used the procedure previously described in [44]. Mice were anaesthetized and perfused via intracardiac infusion with 0.1 M PBS and then 4% paraformaldehyde (PFA, dissolved in 0.1 M phosphate buffer, pH 7.4) 90 min after completion of phase III (choice phase) or 60 minutes after phase II (learning phase) for c-fos and histone H3 acetylation, respectively. Brains were removed, fixed overnight in PFA, and then transferred to 30% sucrose solution and stored at 4°C. Coronal sections were cut at 40 *μ*m thickness on a freezing microtome (Sliding Leica microtome SM2010R, Leica Microsystems) and processed for immunohistochemistry.

For c-fos immunohistochemistry, after a blocking step in 10% NGS and 0.5% Triton X-100 in PBS, free-floating sections were incubated using anti-c-fos rabbit polyclonal antibody (1 : 3000 rabbit anti-c-fos polyclonal antibody, Calbiochem, USA) for 36 h at 4°C. Subsequently, sections were transferred in a solution containing 1% NGS, 0.1% Triton X-100, and 1 : 200 anti-rabbit biotinylated antibody (Vector Labs) in PBS. This was followed by incubation in ABC kit (Vector Labs) and final detection with DAB reaction kit (Vector Labs). Sections were finally mounted on gelatinized slides, dehydrated, and sealed with DPX mounting medium (VWR International, UK).

For acetyl-histone H3 immunohistochemistry, after a blocking step in 10% NGS and 0.05% Triton X-100 in PBS, sections were incubated in a solution containing 10% NGS, 0.05% Triton X-100, and anti-acetyl-histone H3 monoclonal rabbit antibodies (Lys14, 1 : 200 rabbit anti-acetyl-histone H3 monoclonal antibodies, Millipore, USA) overnight at 4°C. Subsequently, sections were transferred in a solution containing 3% NGS, 0.05% Triton X-100, and 1 : 400 Alexa Fluor 568 goat anti-rabbit IgG antibody (Life Technologies) in PBS (Ciccarelli et al., 2013). Sections were then mounted on gelatinized slides with VECTASHIELD (Vector Labs).

### 2.4. Quantitative Analysis of c-fos-Positive Cells

We used the procedure previously described in [44]. Counting of c-fos-positive cells in different brain areas was performed using a CCD camera (MBF Bioscience, Germany) mounted on a Zeiss Axioskop (Zeiss, Germany) microscope and the Stereo Investigator software (MBF Bioscience). Brain structures were anatomically defined according to a mouse brain atlas (Paxinos and Franklin, 1997), and the regions of interest selected for measurement of c-fos-positive nuclei in the orbitofrontal cortex (OFC) were (numbers indicate the distance in millimeters of the sections from bregma) medial orbital cortex (MO, +2.80 mm), ventral orbital cortex (VO, +2.80 mm), lateral orbital cortex (LO, +2.80 mm), and dorsolateral orbital cortex (DLO, +2.80 mm). The number of c-fos-positive cells was counted at 20x magnification, in 5–10 fields (50 × 50 *μ*m or 100 × 100 *μ*m) per section according to the size of brain structure and their density calculated (cells/mm^2^), using at least 5 sections for each structure. The experimenter counting c-fos-positive cells was blind to the rearing condition and treatment of the animals.

### 2.5. Quantitative Analysis of Immunohistochemical Signal of Histone H3 Acetylation in the OFC

The imaging of brain areas was performed using a CCD camera (MBF Bioscience, Germany) mounted on a Zeiss Axioskop (Zeiss, Germany) microscope and QCapture software (QImaging, Canada). Brain structures were anatomically defined according to a mouse brain atlas (Paxinos and Franklin, 1997). Images were acquired at 20x magnification in one field of 200 × 300 *μ*m per section, keeping constant both microscope settings and fluorescence-field intensity. The collected images were imported to the image analysis system MetaMorph (molecular devices), and for each animal, the relative signal intensity of AcH3-immunopositive cells was calculated using at least 5 sections for each structure. The relative signal intensity of AcH3-immunopositive cells was calculated as the ratio between the mean intensity of AcH3-immunopositive cells and the intensity of background signal measured in sample areas surrounding AcH3^+^ cells. All image acquisition and analysis were carried out in blind.

### 2.6. Statistics

All results were expressed as mean ± SEM, and all statistical analyses were performed using statistical software package SigmaStat (SigmaStat, version 3.5). For STFP performance in phase III, a two-way analysis of variance (ANOVA) for repeated measures (RM) was performed for each retention interval (1 h, 24 h, or 30 days), considering both factor condition (EE or SC or PL-EE or PL-SC) and factor flavor (familiar or novel), with post hoc analysis Holm-Sidak method. The number of c-fos-positive cells in each OFC area was analyzed with a two-way ANOVA, factor condition and retention interval, with post hoc analysis Holm-Sidak method. The level of histone H3 acetylation in each OFC area was analyzed with a two-way ANOVA, factor condition, and area, with post hoc analysis Holm-Sidak method.

## 3. Results

### 3.1. Long-Term STFP Memory Deficit in Aged Mice Is Ameliorated by EE

**Figure 1:**
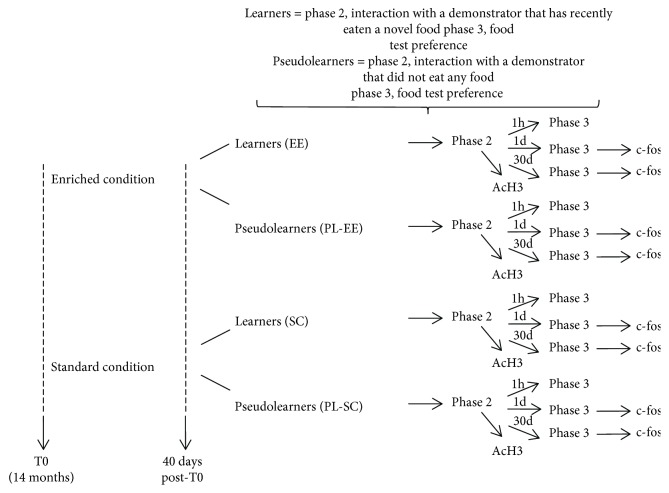
Schematic diagram of the experimental protocol.

Aged C57BL/6 mice, housed in EE or SC for 40 days, were subjected to the STFP task. During the learning phase (phase II), interactions between demonstrator and observer mice were scored to control for possible differences between EE and SC mice in the amount of interactions (a schematic diagram of the experimental protocol is reported in Figure 1).

Indeed, a difference in interaction with the demonstrator during the learning phase would affect performance in the choice phase: an observer mouse is expected not to show any preference for the cued food if it has not adequately interacted with the demonstrator during the learning phase. We found no difference in the amount of interactions between EE and SC mice (*t*-test, EE (*n* = 13) versus SC (*n* = 16), *p* = 0.111; data not shown).

Then, we evaluated olfactory memory abilities in the choice phase (phase III) of the STFP task: in this phase, observer mice, after a retention interval, were placed in individual cages allocating two jars: one jar contained a familiar food identical to that consumed by the demonstrator and the other containing a totally novel food. After 1 h spent in the choice phase, the jars were removed from the cage and weighed in order to assess the amount of food eaten from each jar. The choice phase was performed at three different intervals, that is, 1 h, 24 h (day 1), or 30 days (day 30) after the end of phase II.

**Figure 2:**
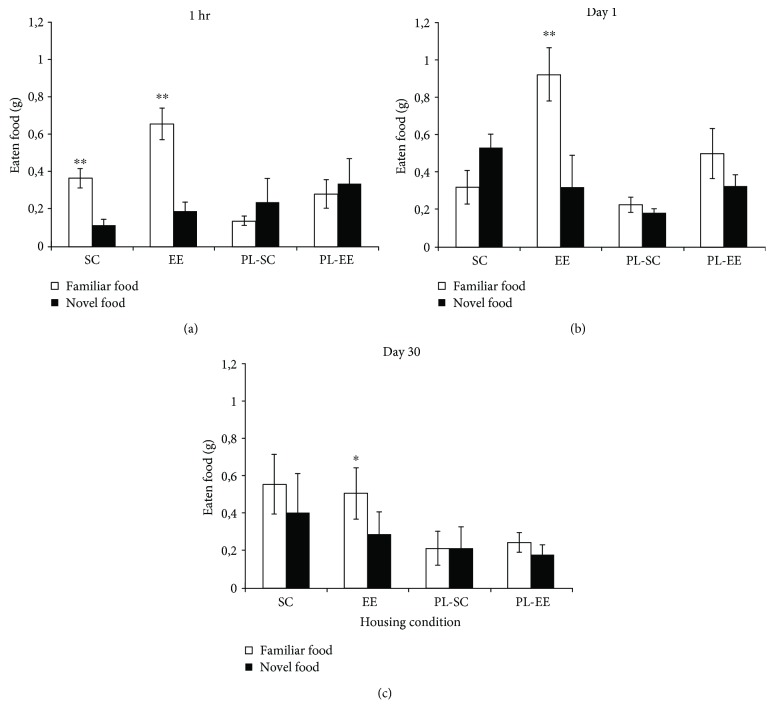
Performance of SC, EE, PL-SC, and PL-EE mice in the STFP. (a) 1-hour retention interval: there is a significant preference for the familiar food (open columns) with respect to the novel food (filled columns) for SC and EE groups, which underwent associative learning of familiar food in the breath of the demonstrator (two-way RM ANOVA post hoc analysis Holm-Sidak method, familiar food versus novel food for SC (*p* = 0.007) and EE (*p* < 0.001)). (b) 24-hour retention interval (day 1). There is a significant preference for the familiar food with respect to the novel food only for EE group (two-way RM ANOVA post hoc analysis Holm-Sidak method, familiar food versus novel food for EE (*p* = 0.001)). (c) 30-day retention interval (day 30): there is a significant preference for the familiar food with respect to the novel food only for EE group (two-way RM ANOVA post hoc analysis Holm-Sidak method, familiar food versus novel food for EE (*p* = 0.021)). There is no preference for the familiar food in pseudolearning mice, PL-SC and PL-EE, for any time interval (two-way RM ANOVA post hoc analysis Holm-Sidak method (*p* > 0.05) for all comparisons). ^∗^*p* < 0.05; ^∗∗^*p* < 0.01; error bars = SEM.

At 1 h retention interval, we found a clear preference for the familiar food in both groups of animals which underwent associative learning of familiar food in the breath of the demonstrator, SC (*n* = 8) and EE (*n* = 5) (two-way RM ANOVA, post hoc analysis Holm-Sidak method, familiar food versus novel food, *p* = 0.007 for SC, *p* < 0.001 for EE; Figure 1); on the contrary, at retention intervals 24 h (day 1) and 30 days (day 30), we found a significant familiar food preference only for EE mice, while SC mice displayed no preference at either interval (two-way RM ANOVA post hoc analysis Holm-Sidak method, familiar food versus novel food at day 1, *p* = 0.153 for SC mice (*n* = 4), *p* = 0.001 for EE mice (*n* = 4); at day 30, *p* = 0.095 for SC mice (*n* = 4), *p* = 0.021 for EE mice (*n* = 4); Figure 2).

As expected, no food preference was displayed by PL-SC and PL-EE mice, which did not undergo associative learning (two-way RM ANOVA, post hoc analysis Holm-Sidak method, novel versus familiar; *p* > 0.05 at both retention intervals; Figure 2).

The good performance of SC mice at 1 h retention interval rules out the possibility of olfactory deficits hampering the preference for the familiar food in aged SC mice. Thus, their failure in eating significantly more the familiar food at day 1 and day 30 indicates a long-term STFP memory deficit in aged SC mice, which is rescued by EE.

### 3.2. c-fos Expression in the OFC of Aged SC and EE Mice following a Retention Interval of 1 or 30 Days

**Figure 3:**
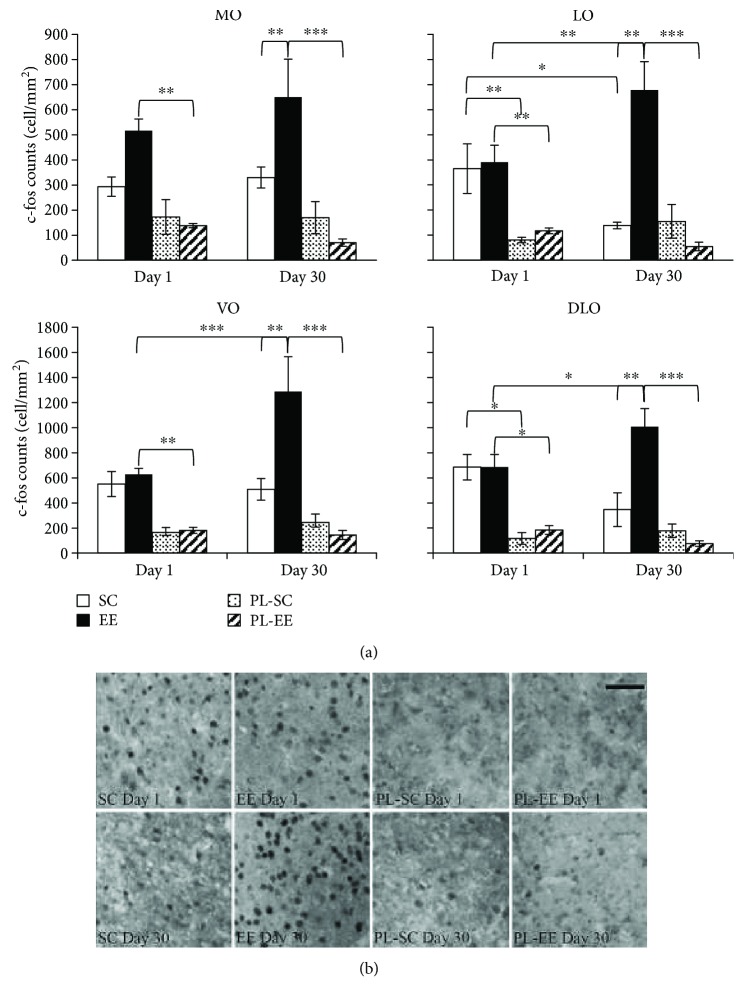
(a) c-fos expression in subregions of OFC for SC (open columns), EE (filled columns), PL-SC (dotted columns), and PL-EE (hatched columns) mice subjected to choice phase at day 1 and day 30. MO: two-way ANOVA, factor condition (*p* < 0.001), factor time (*p* = 0.613), condition × time (*p* = 0.530), post hoc analysis Holm-Sidak method. SC versus PL-SC and PL-SC versus PL-EE (*p* > 0.05) for all retention intervals. Statistical differences were found between EE versus PL-EE at day 1 (*p* = 0.001) and day 30 (*p* < 0.001); EE versus SC significant at day 30 (*p* = 0.003). LO: two-way ANOVA, factor condition (*p* < 0.001), factor time (*p* = 0.688), condition × time (*p* = 0.004), post hoc analysis Holm-Sidak method. PL-SC versus PL-EE (*p* > 0.05) for all retention intervals. Statistical differences were found between SC versus PL-SC at day 1 (*p* = 0.004); EE versus PL-EE at day 1 (*p* = 0.001) and day 30 (*p* < 0.001); EE versus SC significant at day 30 (*p* < 0.001). Statistical differences within group were found between day 1 and day 30 for SC group (*p* = 0.018) and for EE group (*p* = 0.004). VO: two-way ANOVA, factor condition (*p* < 0.001), factor time (*p* = 0.054), condition × time (*p* = 0.015), post hoc analysis Holm-Sidak method. SC versus PL-SC and PL-SC versus PL-EE (*p* > 0.05) for all retention intervals. Statistical differences were found between EE versus PL-EE at day 1 (*p* = 0.001) and day 30 (*p* < 0.001); EE versus SC significant at day 30 (*p* < 0.001). Statistical differences within EE group were found between day 1 and day 30 (*p* < 0.001). DLO: two-way ANOVA, factor condition (*p* < 0.001), factor time (*p* = 0.913), condition × time (*p* = 0.059), post hoc analysis Holm-Sidak method. PL-SC versus PL-EE (*p* > 0.05) for all retention intervals. Statistical differences were found between SC versus PL-SC at day 1 (*p* = 0.003); EE versus PL-EE at day 1 (*p* = 0.002) and day 30 (*p* < 0.001); EE versus SC significant at day 30 (*p* < 0.001). Statistical differences within EE group were found between day 1 and day 30 (*p* = 0.035). ^∗^*p* < 0.05; ^∗∗^*p* < 0.01; ^∗∗∗^*p* < 0.001; error bars = SEM. (b) Representative panel of c-fos protein expression in DLO for SC, EE, PL-SC, and PL-EE mice, following recall at day 1 and day 30; scale bar: 50 *μ*m.

After the end of the choice test at day 1 and day 30, when SC and EE mice significantly differed in their memory performance, mice were sacrificed and the expression of c-fos protein was assessed as an indicator of neuronal activity in the OFC, the final olfactory memory storage site. c-fos immunohistochemistry is currently used in developing adult and aging animals as a reliable, surrogate marker for neuronal activity, particularly when spatial distribution of neuronal activity and comparison of neural activation between different brain regions is of interest (see, e.g., [10, 44–50]). We separately counted c-fos-positive cells in 4 OFC subregions, medial orbital cortex (MO), ventral orbital cortex (VO), lateral orbital cortex (LO), and dorsolateral orbital cortex (DLO). No difference was present between the two control groups, PL-EE and PL-SC mice, in any OFC region at any retention interval (two-way ANOVA, post hoc analysis Holm-Sidak method, PL-EE versus PL-SC, *p* > 0.05; Figure 3), suggesting that EE and SC condition per se does not affect c-fos expression in the areas of interest during the choice test.

At day 1, we found significant neuronal activation in OFC both for EE and SC mice: indeed, the number of c-fos-positive cells was higher in SC and EE mice with respect to their controls (PL-SC and PL-EE mice) in all OFC areas in EE mice and in LO and DLO in SC mice (two-way ANOVA, post hoc analysis Holm-Sidak method for condition, EE versus PL-EE (*p* < 0.01) for all areas; SC versus PL-SC (*p* < 0.01) for LO and DLO; SC versus PL-SC (*p* > 0.05) for MO and VO; Figure 3). No difference was present between EE and SC mice in any OFC regions (two-way ANOVA, post hoc analysis Holm-Sidak method for condition, EE versus SC (*p* > 0.05) for all areas; Figure 3).

At day 30 retention interval, we found significant neuronal activation only in EE mice: in particular, we found an increased number of c-fos-positive cells in all OFC areas of EE mice (two-way ANOVA, post hoc analysis Holm-Sidak method for condition, EE versus PL-EE (*p* < 0.001) in all areas; Figure 3). No increase in the number of c-fos-positive cells was present in any OFC area at day 30 in SC mice with respect to their controls (two-way ANOVA, post hoc analysis Holm-Sidak method for condition, SC versus PL-SC (*p* > 0.05) in all areas; Figure 2), indicating lack of neuronal activation. A comparison between EE and SC c-fos expression at day 30 showed a greater neuronal activation in EE with respect to SC mice in all OFC areas (two-way ANOVA, post hoc analysis Holm-Sidak method for housing condition, EE versus SC, *p* = 0.003 for MO and *p* < 0.001 for VO, LO, and DLO; Figure 3).

In the comparison between c-fos activations at day 1 and day 30, we found a significantly higher number of c-fos-positive cells at day 30 with respect to day 1 in EE mice in all OFC areas but MO (two-way ANOVA, post hoc analysis Holm-Sidak method 30 versus 1 in EE, *p* = 0.181 in MO; *p* < 0.001 in VO; *p* = 0.004 in LO; *p* = 0.035 in DLO; Figure 3); on the contrary, no significantly higher activation of OFC areas was found at day 30 with respect to day 1 in SC mice; rather, we found a decrease in c-fos expression in LO for the SC group (two-way ANOVA, post hoc analysis Holm-Sidak method 30 versus 1 in SC, *p* = 0.018 in LO; *p* > 0.05 in MO, VO, and DLO; Figure 3).

Thus, preserved memory at day 30 in EE mice was associated with a significant activation of OFC and with increased OFC activation with respect to day 1 recall.

### 3.3. Increased Histone H3 Acetylation in the OFC of EE Aged Mice 1 h after the Learning Phase

Epigenetic changes are crucially involved in memory consolidation [35, 51]. Lesburgueres et al. [36] have shown that the setting of synaptic tags during system consolidation of STFP memory involves epigenetic mechanisms, in particular increased acetylation of histone H3 in the OFC shortly (1 h) after the learning phase; preventing the increase in H3 acetylation impaired remote memory, assessed 30 days later, while pharmacological maintenance of a higher level of acetylation resulted in improved remote memory retrieval. Using Lesburgueres et al. protocol, we have assessed AcH3 levels in OFC 1 h after the learning phase.

**Figure 4:**
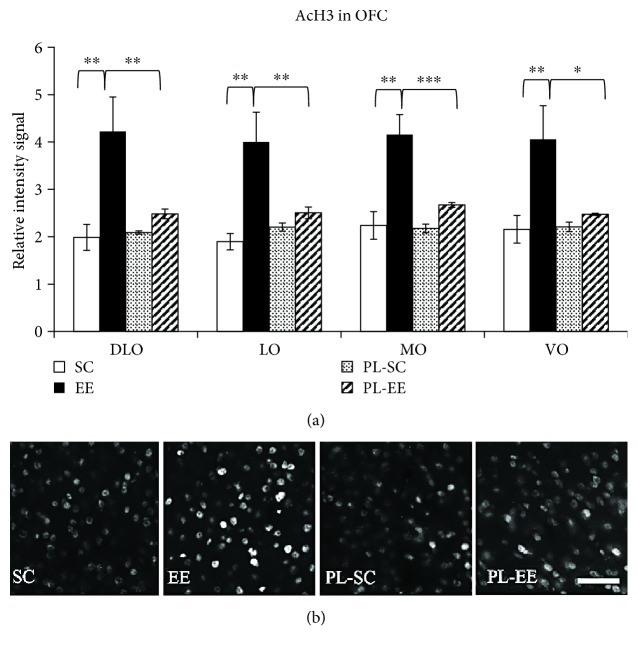
(a) H3 acetylation on L14 in subregions of OFC for SC (open columns), EE (filled columns), PL-SC (dotted columns), and PL-EE (hatched columns) mice 1 hour after the learning phase. PL-SC did not differ from PL-EE mice. Only EE animals showed significant differences with respect to their controls in OFC areas; (one-way ANOVA (*p* < 0.01), post hoc analysis Holm-Sidak method, SC versus PL-SC and PL-SC versus PL-EE (*p* > 0.05) for all subregions). MO: EE versus SC (*p* < 0.001), EE versus PL-EE (*p* = 0.001). LO: EE versus SC (*p* < 0.001), EE versus PL-EE (*p* = 0.007). VO: EE versus SC (*p* = 0.004), EE versus PL-EE (*p* = 0.013). DLO: EE versus SC (*p* = 0.001), EE versus PL-EE (*p* = 0.008). ^∗^*p* < 0.05; ^∗∗^*p* < 0.01; ^∗∗∗^*p* < 0.001; error bars = SEM. (b) Representative panel of histone H3 acetylation in DLO for SC, EE, PL-SC, and PL-EE mice 1 h after learning; scale bar: 50 *μ*m.

We found that the signal intensity of histone H3 acetylation on lysine 14 was significantly increased with respect to control PL mice only in EE mice, and this was found in all subregions of OFC (one-way ANOVA, *p* < 0.01, post hoc analysis Holm-Sidak method, EE versus PL-EE, *p* < 0.01 for all subregions; Figure 4); no intensity difference was found between SC and PL-SC or between PL-EE and PL-SC mice (one-way ANOVA, *p* < 0.01, post hoc analysis Holm-Sidak method, SC versus PL-SC, *p* > 0.05 for all subregions; Figure 4). Intensity of H3 acetylation in EE mice was significantly higher than in SC mice in all OFC regions (one-way ANOVA, *p* < 0.01, post hoc analysis Holm-Sidak method, EE versus SC, *p* < 0.01 for all subregions; Figure 4).

Thus, the preserved remote memory recall in aged EE mice is associated with enhanced OFC histone acetylation early in the system consolidation process, which suggests a preserved early tagging of OFC.

## 4. Discussion

Brain aging is a complex physiological process characterized by a progressive deterioration of cognitive functions, especially in the learning and memory domains. Major deficits are particularly evident at the level of declarative memory abilities requiring the precise recall of detailed information, a process initially mediated by the hippocampus and other medial temporal lobe structures [1, 4] and subsequently supported by the process of system consolidation. System consolidation consists in the functional and structural reorganization of cerebral regions prompted by the reactivation of hippocampal-cortical pathways and the strengthening of corticocortical connections, leading to formation of a cortical memory trace which supports remote memory recall [36, 52]. Many papers have investigated the effects of EE on local memory consolidation in the aged hippocampus, responsible for formation and early maintenance of hippocampus-dependent memory; for instance, EE ameliorates CA1 plasticity and cell excitability and reverses age-related epigenetic changes and other age-related negative processes (see, e.g., [20–22, 31, 53]). However, to our knowledge, the effects of EE on system consolidation, that is, on the recruitment of cortical activity to support remote memory, have not been investigated in aged animals. In particular, we found no study on the effects of EE on the early tagging in prefrontal cortex as a correlate of remote memory retrieval.

In this study, we showed that exposure to EE in aged mice modulates system consolidation and enhances performance in the STFP declarative memory task. STFP is a hippocampus-dependent task which exploits the natural tendency of rodents to prefer food sources based on a previous sampling of their odor in the breath of littermates. In this task, the behavioral performance can be efficiently measured in a single trial session and, given that the underlying memory traces are long-lasting, it allows researchers to perform a characterization of possible changes in the expression levels of potential transcription factors involved in memory acquisition and in the recall phases. Olfactory memories activated by STFP are eventually transferred to the OFC, via a system consolidation process which requires early tagging of cortical networks in this structure [36].

While early tagging of cortical networks has been previously investigated in adult rodents, no evidence for similar mechanisms has been provided, so far, in aged animals. We first report that, while aged SC animals displayed severe deficits in memory recall at both recent (1 day) and remote (30 days) time intervals, EE preserved cognitive performance in the STFP task at both intervals. The performance of aged EE mice thus resembles that of standard-reared young adult rats, performing well both recent and remote STFP memory recall [36].

Focusing on c-fos expression as a marker of neuronal activity, we analyzed the activation of the final recipient of SFTP memory, namely, OFC, during recent and remote recall. A marked environment-dependent effect was found in the OFC, with enriched mice displaying higher c-fos expression levels compared to SC animals at the 30-day interval, in agreement with the differences in recall abilities displayed by enriched versus SC animals in the SFTP declarative memory task at this time point. Importantly, the absence of OFC activation displayed by SC aged mice at the 30 days of retention interval was not dependent on general olfactory discrimination deficits, as demonstrated by the intact performance displayed by the same group at shorter intervals (1 h). Comparable amounts of OFC activation were instead found in SC and EE animals at the 1-day retention interval, when both groups displayed increased c-fos expression compared to their respective controls; however, this activation of OFC areas was paralleled by marked behavioral deficits evident at this time point. These results indicate that OFC activation recorded 24 h past the end of the acquisition phase is not sufficient for, or not correlated with, mouse mnemonic performance. This is in accordance with the late role for OFC in system consolidation and its dispensability for recent memory recall [36, 52, 54, 55].

Looking for possible epigenetic changes acting as an early tagging mechanism for memory formation in olfactory cortical networks, we found an increased H3 acetylation in all analyzed OFC areas of EE subjects compared to SC animals. H3 acetylation levels were found to be enhanced with respect to control animals in EE mice, but not in SC animals. These results suggest a deficit in the initial steps of cortical tagging of memory traces associated with brain aging in SC compared to EE animals and demonstrate that exposure to stimulating environmental conditions preserves remote recall of declarative memory abilities in aged mice by promoting system consolidation through the activation of epigenetic regulatory processes crucial for coding and consolidation of cortical mnemonic traces. System consolidation is the process of time-dependent gradual reorganization of the brain regions supporting remote memory storage. Within this process, early tagging of cortical circuits is a necessary step for the formation of enduring associative memory [35, 36]. In particular, early tagging of OFC cortical networks is a prerequisite for the establishment of remote olfactory memory of the STFP. Synaptic tags may serve as an early and persistent signature of activity in the cortex that is necessary to ensure the progressive rewiring of cortical networks that support remote memory storage. The early increase in histone H3 acetylation following STFP learning phase is involved in the formation of the early synaptic tags in OFC supporting STFP remote memory retrieval: preventing the increase in H3 acetylation impaired remote memory, assessed 30 days later, while pharmacological maintenance of a higher level of acetylation resulted in improved remote memory retrieval probed 30 days later [36]. Our results show that in the absence of H3 acetylation following learning, as is the case of aged SC mice, there is a strong memory deficit. EE ameliorates memory performance in aged mice, allowing a good retrieval of memory 30 days after learning and this is associated with an increased H3 acetylation in OFC of EE mice. This suggests that EE preserves a good early tagging in aged mice and strengthens the hypothesis that early tagging is important for system consolidation.

It has been previously reported that an increased activity of histone acetyltransferase (HAT) enzymes during a hippocampus-dependent task can be accompanied by increased histone acetylation acting as a specific epigenetic tagging for memory consolidation [56]. It is well known that environment-induced beneficial effects on brain plasticity and memory abilities may involve HAT activation [9, 28]. The increased AcH3 found in our EE mice might suggest an involvement of HAT activation; however, further work would be necessary to elucidate the mechanisms underlying this increased acetylation.

At the 24 h retention interval, OFC activation in SC mice was enhanced with respect to their PL controls, reaching levels not significantly different from those displayed by EE mice; however, differently from EE mice, in SC mice, OFC activation at the 24 h of retention interval was not preceded by any early tagging process, suggesting that it is the lack of early tagging and not the lack of general recruitment of OFC areas that is responsible for the main deficits in remote associative memory formation displayed by SC aged mice. Indeed, the crucial and irreplaceable role of early tagging is that of topographically specifying, by means of epigenetic signature [35, 36], which sets of cortical neurons will participate to the hippocampal-cortical dialogue during the course of systems-level consolidation. In the absence of early tagging, that is, of the specification of the recipient circuits in OFC, the general activation of OFC would be unlikely to sustain the fine process of transferring specific information from the hippocampus to OFC to sustain formation of enduring associative memory.

EE is well known to affect synaptic function not only in young or adult but also in aged animals, where EE ameliorates many of the deficits in synaptic physiology, density, and plasticity induced by age (see [1, 9, 20, 22]). We cannot exclude that these effects of EE might have contributed to our results, for instance, via a better encoding/local consolidation process in the hippocampus, especially in the case of the better performance of EE animals at the 24 h interval, where early tagging in PFC is not supposed to play a key role; enhanced synaptic function and plasticity in EE animals might also have contributed to our results enhancing the efficacy of the hippocampal-cortical dialogue during formation of the cortical trace which supports remote memory.

In conclusion, our results show that exposure to stimulating environmental conditions can be used as a powerful paradigm to promote good system consolidation of associative memory in aged mice and suggest that system consolidation may be a crucial target for treatments aimed at ameliorating memory dysfunctions in elderly subjects.

## Data Availability

The data used to support the findings of this study are available from the corresponding author upon request.
